# Effects of a Physical-Literacy-Based Educational Intervention on Physical Activity and Body Composition in Preadolescent Children: A School-Based Controlled Trial

**DOI:** 10.3390/sports14020077

**Published:** 2026-02-09

**Authors:** Petra Rajkovic Vuletic, Barbara Gilic, Vladimir Pavlinovic, Paula Matijasevic, Damir Sekulic

**Affiliations:** 1 Faculty of Kinesiology, University of Split, 21000 Split, Croatia; petra.rajkovic@kifst.eu (P.R.V.); barbara.gilic@kifst.eu (B.G.); vladimir.pavlinovic@kifst.eu (V.P.); paula.matijasevic@kifst.eu (P.M.); 2 Faculty of Kinesiology, University of Zagreb, 10000 Zagreb, Croatia

**Keywords:** health education, child, curriculum, physical education, physical activity levels

## Abstract

Improving physical literacy (PL) is recognized as a potentially effective approach for enhancing overall physical activity (PA) and fitness status, including body composition, but few studies have examined the impact of PL-oriented education on directly measured PA and body composition in children. The aim of this study was to evaluate the effects of a three-month quasi-experimental PL-based educational intervention, delivered as part of the regular physical education (PE) curriculum, in preadolescent children. A total of 119 children aged 9–11 years (51 girls) from southern Croatia participated in the study and were assigned to either a control group (*n* = 68) or an intervention group (*n* = 51). The intervention group received a PL-focused educational program integrated into regular PE classes, whereas the control group followed the standard PE curriculum. PA was assessed using accelerometers, and body composition was measured using bioimpedance analysis. A pre–post–retention design was applied (baseline at the start of the school year, post-intervention in December, and retention testing at the end of the school year), and a three-way repeated-measures ANOVA (group × gender × time) was conducted. The intervention prevented declines in vigorous physical activity (VPA) and step count (STEPS) and maintained overall sedentary time (ST) in the experimental group throughout the school year. Significant group × time interaction effects were detected for VPA, STEPS, and ST (F = 4.01, 4.09, and 5.34, respectively; all *p* < 0.05). No significant effects were found for body composition. In conclusion, the PL-based intervention allowed effective mitigation of the typical seasonal decline in activity levels observed during the school year. Further studies evaluating the effects of similar interventions on other indices of fitness status are warranted.

## 1. Introduction

Physical fitness (PF) is a state of health and well-being and, more specifically, refers to the ability to perform aspects of sports, occupations, and daily activities [1]. Optimal PF forms the foundation of a healthy lifestyle and is one of the key indicators of both current and future health status. In this sense, childhood and adolescence are especially important, as high PF levels at these ages directly and positively influence health outcomes in adulthood [2,3,4,5]. Longitudinal studies have shown that maintaining proper PF in children and adolescents reduces the risk of obesity, cardiovascular diseases, and diabetes in adulthood [6,7,8]. Additionally, research on mental health indicates a positive association between PF and general mental health in children and youth [9,10]. However, despite its importance, PF levels among children and adolescents have been steadily declining [11,12].

It is widely accepted that reaching optimal levels of physical activity (PA) is fundamental in achieving and maintaining optimal physical fitness (PF) [13]. However, it is also well known that PA and PF are closely interconnected and influence one another. Research on children and adolescents has confirmed that PA is the primary mechanism for developing and maintaining key PF components such as body composition, cardiorespiratory function, and muscular strength, with high-intensity PA being the most important in exerting the most significant effect on PF [14]. On the other hand, higher PF levels enable children to participate in vigorous PA more easily and for longer periods, as they can withstand greater workloads and sustain high intensity for extended time frames [14,15,16,17]. Research has demonstrated that childhood and adolescence are the most critical phases for establishing such positive cycles between PA and PF [18,19,20].

In general, the prevailing opinion is that increases in PA result in improvements in various abilities among children, including attention, concentration, and different PF capacities [21,22,23,24]. Specifically regarding PF, a study conducted on US children aged 9–11 years reported that PA is positively associated with all components of PF [21]. Similar results were obtained in Spanish children aged 8–12 years, confirming that children with higher levels of PA achieve better PF [22]. Similarly, a Danish study with children aged 8–10 years showed that children involved in sports activities and had higher PA demonstrated better cardiorespiratory capacity, faster sprint times, better coordination, lower body fat indices, and a more favorable cardiovascular profile than their inactive peers [23]. Estonian authors longitudinally followed children from kindergarten to 11–12 years for six years and reported that 73% of children who were continuously physically active achieved healthy levels of PF, whereas only 24% of inactive children did [25]. Finally, a recent Polish study revealed positive effects of increased hours of physical education (PE) on PF in 11- to 12-year-old children [26]. Finally, higher levels of PA, particularly of moderate-to-vigorous intensity, are associated with favorable changes in body composition among children, including reductions in fat mass and improvements in lean mass [27,28].

However, despite the evidence for a positive association between higher PA and better PF in children, a growing body of evidence indicates that simply increasing PA is not always sufficient to improve PF in children, highlighting the fact that emphasis should be placed on promoting specific forms of exercise and targeting those intensities of PA that directly increase PF levels [14,29,30,31]. Given the established intercorrelations between PA and PF, the concept of “physical literacy,” which encompasses a holistic approach to their development, has attracted increased attention over the last decade.

Physical literacy (PL) is defined as the ability of an individual to have the motivation, confidence, and physical competence to engage in PA and to understand the health benefits of PA to develop habits and stay active throughout life [32,33,34]. Research shows that improving PL has beneficial effects on PA and PF in children and adolescents [35,36,37]. For example, a five-month intervention consisting of structured play and active games resulted in positive changes in PA and reduced sedentary behavior in children aged 6–9 years [38]. Furthermore, an eight-week intervention based on a pedagogical approach aimed at developing PL had significant positive effects on balance, stability, and cardiorespiratory endurance in children aged 8–12 years [39]. Similarly, a six-month program aimed at developing PL showed encouraging results in terms of body composition variables in children aged 5–12 years [40]. Positive effects on the PA and PF of 9- to 11-year-old children were evidenced in a twelve-week intervention targeting PL improvement, which included physical education classes conducted by a specialized teacher [41]. Although not all studies reported positive effects of PL improvement on PF and/or PA collectively, it seems that interventions integrating various domains of PL are beneficial in children of younger school age (preadolescents) [42,43,44].

The previous literature overview clearly demonstrates that studies mostly indicate positive effects of PL interventions on PA and PF. However, it is important to emphasize certain limitations of the studies performed to date. First, many of the studies did not include a control group of participants, meaning that the changes reported could be a result of growth and development and/or regular PE and sport participation, and not the PL-based intervention [35,37]. Second, studies regularly applied pre- and post-study designs, and to the best of our knowledge, none of the studies have observed retention of the intervention effects [35,37]. Furthermore, there is an evident lack of studies that have evaluated the changes in anthropometric/body composition of children as a result of PL-intervention. However, this information is highly important because body composition status in childhood may indicate later risks for overweight and obesity [45]. Finally, evaluations of PA levels have been conducted primarily using questionnaires and have rarely used direct measurements of PA [33,35,37].

Therefore, with the intention of improving knowledge in the field, this study aimed to evaluate the effects of one specific PL-based intervention on (i) objectively measured PA and (ii) body composition indices in 9- to 11-year-old children from Croatia, observing PA indices as primary, and body composition parameters as secondary outcomes. As a secondary outcome, the eventual changes in body composition indices as a result of the applied intervention were observed, without a priori expectation of significant effects. We hypothesized that a PL intervention would yield positive results in terms of PA indices.

## 2. Materials and Methods

### 2.1. Participants

The sample in this quasi-experimental school-based controlled trial consisted of 119 preadolescent children (51 girls) from southern Croatia. The sample size calculation was based on the planned method for assessing effects (analysis of variance), the number of planned groups (two participant groups), statistical power (0.8), and the expected effect size (medium). Using an F-test as the type of effect, the required size for a balanced sample (equal number of participants in both groups) was determined to be a total of 108 students across the two groups, yielding a statistical power of 0.811.

At the time of data collection, the participants were between 9 and 11 years old and were enrolled in either the 3rd (*n* = 71) or 4th grade (*n* = 48) of elementary school. All the children were in good health and attended physical education (PE) classes on a regular basis. Those who had been ill or who had sustained a musculoskeletal injury within two weeks prior to testing were excluded. Before participation, parents or legal guardians were informed about the study objectives and procedures, and parental written consent was obtained. The research protocol was preapproved by the Ethics Committee of the Faculty of Kinesiology, University of Zagreb. Most of the participants participated in out-of-school sport activities (78% and 74% in the control and intervention groups, respectively), with no significant difference noted between the groups (Chi square = 1.44, *p* > 0.05). Initially, pubertal timing was estimated for all children using peak height velocity (PHV), as described by Moore et al. (PHV = −7.999994 + (0.0036124 × age (yrs.) × height (cm)), and no difference was found between the C and E groups (*t*-test = 0.23, *p* > 0.05), indicating a similar level of maturity status in both study groups [46].

**Figure 1:**
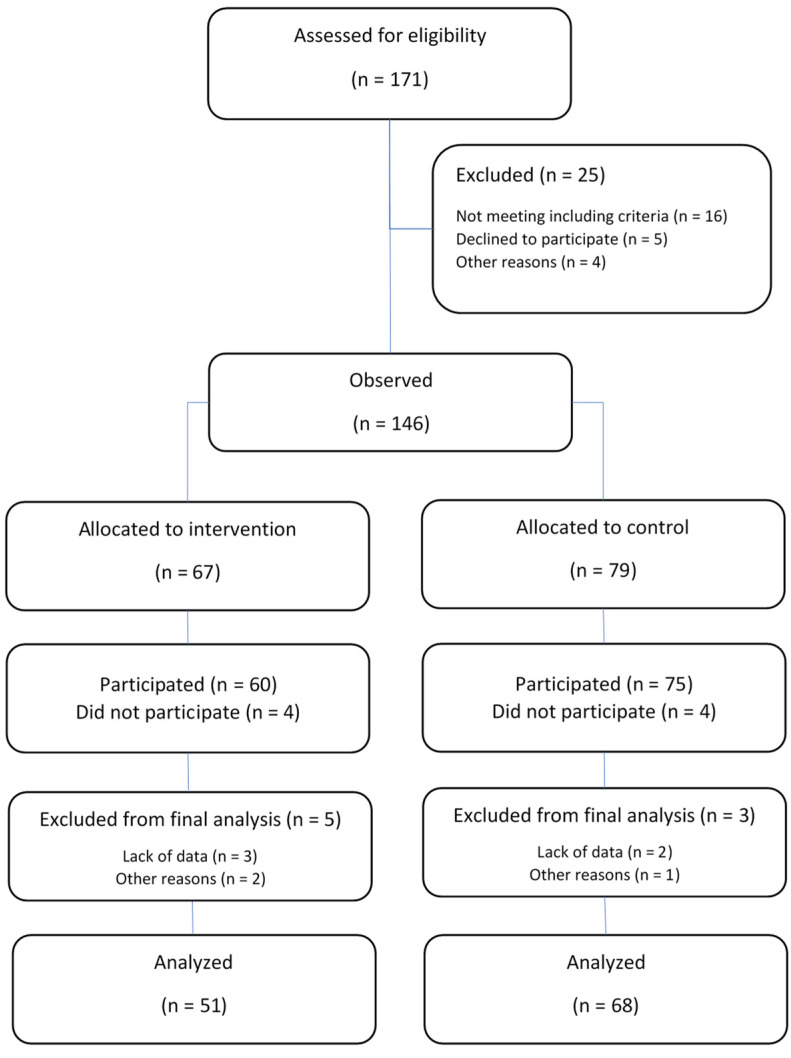
Flow diagram of the study.

The total sample was divided into a control group (C, *n* = 68) and an experimental group (E, *n* = 51). Owing to the nature of the school setting and the fact that the intervention was delivered during PE classes (please see later for details), a quasi-experimental design was employed. Specifically, entire classes, rather than individual students, were assigned to either the experimental group (two classes) or the control group (two classes), resulting in nonrandomized allocation of participants. Given the very small number of clusters, the possible confounding effects of non-randomized grouping, multilevel modeling was not applied (please see later for Statistics), as previous school-based studies have found that intra-class correlations (ICCs) for objectively measured physical activity outcomes in this age group are typically low (e.g., ICC < 0.05), suggesting a limited influence of clustering on the results [47]. A flow diagram of the study is presented in Figure 1.

### 2.2. Variables

In addition to age (in years), gender (male vs. female), and school grade (3rd vs. 4th), we directly measured PA and anthropometrics/body composition in this study.

Physical activity was objectively measured using GENEActiv triaxial accelerometers (Activinsights Ltd., Cambridge, UK). This compact (36 mm × 30 mm × 12 mm), lightweight (16 g), waterproof device is equipped with a seismic acceleration sensor and records body temperature, which enhances the accuracy of energy expenditure estimation and the detection of nonwear periods. The GENEActiv accelerometer is widely validated and commonly used for the assessment of physical activity, sedentary behavior, and sleep patterns in both research and clinical settings [48,49].

The participants were instructed to wear the accelerometer continuously on their nondominant wrist for 24 h per day throughout the monitoring period. Raw acceleration data were downloaded using GENEActiv PC software (version 2.2) in .bin format and processed with the GGIR package in R (version 1.2-0). This software automatically detects non-wear time and classifies activity intensities using Euclidean Norm Minus One (ENMO) metrics, following validated acceleration thresholds developed specifically for wrist-worn GENEActiv devices in pediatric populations [50]. The following cut-points were applied by default within the GGIR algorithm: (i) light physical activity (LPA): 35–200 mg, moderate-to-vigorous physical activity (MVPA): >200 mg, vigorous physical activity (VPA): >707 mg. These thresholds were not manually defined by the researchers but were implemented automatically by the GGIR software to ensure consistency and reproducibility. The analysis yielded estimates for total number of steps performed (STEPS; count per week), sedentary time (ST; not including sleep time; min/week), and time spent in light (LPA; min/week), moderate-to-vigorous physical activity (MVPA; min/week), and vigorous physical activity (VPA; min/week). GENEActiv devices record continuous raw triaxial acceleration, eliminating the need for predefined epoch lengths. The GGIR algorithm was used to automatically detect nonwear periods and classify activity intensities on the basis of established GENEActiv-specific thresholds. Participants with insufficient valid wear time were excluded from the final analysis. To be included, individuals were required to have at least four valid days (24 h), including a minimum of two weekdays and two weekend days, in accordance with best practices for measuring habitual PA in children. Later, a weekly average was calculated based on measured data, summarizing weekday and weekend values to represent the entire week. Importantly, activity intensity metrics (LPA, MVPA, and VPA) were derived directly from raw acceleration data using the GGIR algorithm, avoiding the need for user-defined cutoff points.

Anthropometric and body composition measurements were collected in the morning, at least three hours after waking and following normal food and fluid intake. Body height was measured in centimeters with a stadimeter. Body mass and composition were assessed using a bioelectrical impedance analyzer (Tanita TBF-300, Tokyo, Japan) in accordance with established guidelines. A 0.2 kg correction was applied to account for clothing weight. The participants’ age, sex, and height were entered prior to measurement. Height was measured using a stadiometer (Seca, Birmingham, UK). During the measurement, the participants stood barefoot, with their hands placed on the designated electrodes. In addition to body height (cm) and mass (kg), the variables obtained included the calculated body mass index (BMI, kg/m^2^), fat mass (FM, in % of body mass), and skeletal muscle mass (SMM, in % of body mass). All the measurements for all the participants were identical, organized by schools, and conducted by trained personnel. The same measurement protocols, staff, and devices were used at all three time points (see later for details on the study protocol) to ensure measurement reliability. The applied measurement protocols and instruments were already applied to similar samples and checked for validity, and details are available elsewhere [51].

### 2.3. Intervention and Protocol of the Study

**Figure 2:**
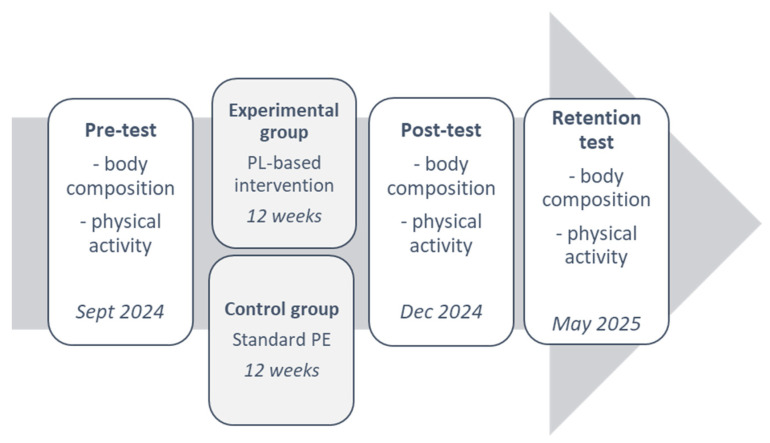
Study protocol.

All participants were tested on study variables at pre-test (September 2024), post-test (late December 2024), and retention (May 2025), with the intervention period occurring between pre- and post-measurement. The study protocol is presented in Figure 2.

The PL-focused educational intervention delivered to the E group was embedded within regular PE over a period of three months (12 weeks). During this time, students had three PE lessons per week, resulting in a total of 36 PE lessons across the intervention period. The PL intervention consisted of 12 original educational video materials, each lasting 3–4 min, and addressed different components of PL and PH. Specifically, the PL theme included 2 videos, covering the cognitive domain, focusing on knowledge and understanding, as well as the affective domain, addressing motivation, self-confidence, and self-efficacy. The Cardiovascular Endurance theme was covered by 3 videos. These videos addressed theoretical knowledge of the topic, and provided practical examples (i.e., how to perform cardiovascular activities) and the recommended amount of physical activity necessary to produce positive effects on this capacity. The strength theme was covered by 3 videos, focusing on cognitive and affective learning, along with practical examples that explain how to build strength, the recommended duration and frequency of exercises, and how to use equipment that is commonly available at home. The flexibility theme featured 2 videos, addressing this capacity from the cognitive and affective perspectives, and included specific examples that demonstrate different types of stretching exercises and correct execution techniques. The final topic was named “healthy habits and nutrition” and contained 2 videos, emphasizing the importance of healthy eating and hydration, and their roles in supporting physical activity and overall well-being. In general, these educational videos were designed to support a deeper understanding of PL concepts through both cognitive insights and practical applications. By combining theory with accessible demonstrations, the authors tried to enhance teaching and learning in diverse settings. All videos are freely available, and the authors hope they will assist educators in their efforts to promote physical literacy and healthy lifestyles around the world. Each video was shown three times during the program to support reinforcement and retention of key messages (please see Supplementary Materials for videos).

The main idea was to provide brief yet clear explanations of PL in general, cardiovascular endurance, and each targeted motor ability, highlighting its relevance in daily life. Moreover, the videos emphasized that physical abilities can be developed using simple, self-directed activities that do not require structured environments. Children were given practical guidance on how to use home-available equipment such as ropes, stairs, water-filled bottles, or carpets used as training mats as substitutes for gym equipment, which was backgrounded in previous knowledge that PE curriculum in Croatia could be broadened by incorporating elements that allow students to develop the knowledge and confidence to perform PA and exercise outside of class [52]. By reinforcing the idea that movement opportunities exist beyond formal PE or sports training, the intervention aimed to empower children to recognize and utilize their own environments to stay active.

We decided to include the video-based instruction because it is increasingly recognized as an effective pedagogical tool for enhancing knowledge acquisition, particularly among children. By combining visual, auditory, and contextual learning cues, videos support multiple learning styles and promote better retention compared to text-based materials alone [53]. Moreover, short and focused educational videos have been shown to maintain attention and facilitate understanding, especially when followed by guided discussion or active reflection [54]. In the context of PE, video content can vividly demonstrate movement concepts and explain their real-life applications. As a result, video education could model desirable behavior and increase physical activity. Indeed, PL-based video instructions were shown to be effective in adolescents during the COVID-19 pandemic period [36]. In this study, brief, thematically structured videos were employed to reinforce the most important PL concepts using minimal resources and within a limited instructional timeframe.

The videos were accompanied by simultaneous explanations and teacher-led discussions, helping students engage with the content in a meaningful way. To maintain instructional balance, each video and discussion substituted approximately 5–7 min of regular PE activity time (within the standard 45 min lesson), allowing the intervention to be seamlessly integrated without compromising the overall structure of the PE curriculum. In brief, in Croatian primary schools, a typical PE lesson is structured into three main parts (i) intro (5–10 min; including light warm-up, dynamic exercises, basic movements, and short games or coordination and attention drills), (ii) main part (30 min; focused on developing motor skills and abilities, technical and tactical elements of sports, and team-based or individual physical activities, and (iii) final part (5–10 min; cool-down activities, breathing exercises, and stretching; short reflection, feedback, or motivational discussion). The educational videos were delivered as the final part of the PE classes, and therefore did not interfere with standard PE themes, meaning that standard motor practices did not differ between study groups. During the study period, the C group participated in the same number of PE lessons (36 total), but their curriculum followed the standard national PE program, which included traditional physical education themes without the PL-educational video content. The intervention summary is presented in Table 1.

Structured procedures were implemented throughout the study period. All teachers involved in the intervention group received standardized training prior to implementation. Initial training sessions were conducted separately at each school by the first authors, who were also the designers of the video materials. These sessions covered the conceptual background of physical literacy (PL), instructional goals for each video, recommended classroom prompts, and clarification strategies. Also, possible questions and answers were covered. Weekly briefings continued throughout the 12-week period to align teaching practices and ensure pedagogical coherence. Fidelity of implementation was monitored via random, non-intrusive classroom observations conducted by the first author and through internal supervision by school officials. Teachers were instructed not to deviate from the established delivery format and to integrate the video content into the final segment of each PE lesson. After viewing each video, students were asked to summarize key concepts, allowing teachers to detect and correct misunderstandings, per initial training instructions. Student adherence to the intervention was monitored using school attendance logs, and only participants who attended at least 80% of the PE sessions were included in the final analysis. The standardization, fidelity monitoring, and protocol details are given in Table S1 (Supplementary Files).

The study protocol was registered at ISCRTN (The UK’s Clinical Study Registry; ISRCTN49955797; 1 September 2024).

### 2.4. Statistics

All variables were tested for normality using the Kolmogorov–Smirnov test. Descriptive statistics are presented as means and standard deviations for continuous variables and as frequencies for categorical variables (gender and grade).

A 3 × 2 × 2 mixed-design ANOVA was conducted to examine the effects of time (pre-, post-, and retention intervention; within-subjects factor), group (E vs. C), and gender (male vs. female; between-subjects factors) on each dependent variable. Box’s M test was used to assess the homogeneity of covariance matrices, and Levene’s test was used to evaluate the equality of error variances. Cell sizes were assessed to ensure approximate balance across conditions.

Effect sizes were reported using partial eta squared (η^2^) for main and interaction effects and interpreted using conventional thresholds as follows: small (η^2^ = 0.01–0.06), medium (η^2^ = 0.061–0.14), and large (η^2^ ≥ 0.141). For significant effects, post hoc analyses were conducted using Scheffé’s test. To reduce the risk of Type I error due to multiple comparisons, a Bonferroni correction was applied where appropriate.

All statistical analyses were performed using Statistica version 14.5 (TIBCO Inc., Palo Alto, CA, USA), with a significance level set at *p* < 0.05.

## 3. Results

The main and interaction ANOVA effects are presented in Table 2. Significant main effects for “group” were found for STEPS (η^2^ = 0.03, small ES), ST (η^2^ = 0.03, small ES), and VPA (η^2^ = 0.03, small ES). The main effect for “gender” was significant for STEPS (η^2^ = 0.15, large ES) and VPA (η^2^ = 0.12, large ES). When the main effect “time” was observed, statistical significance was reached for height (η^2^ = 0.84, large ES), mass (η^2^ = 0.11, moderate ES), SMM (η^2^ = 0.09, moderate ES), STEPS (η^2^ = 0.03, small ES), and VPA (η^2^ = 0.12, moderate ES). The “time × group” interaction effect was significant for STEPS (η^2^ = 0.03, small ES), ST (η^2^ = 0.03, small ES), and VPA (η^2^ = 0.04, small ES), indicating differential changes in the E and C groups over the course of the study. No significant Time × Group interactions were observed for body composition indices.

Descriptive statistics for the total sample and the significance of post hoc differences for the ANOVA main effect “time” are presented in Table 3. Body height and mass increased during the course of the study, with significant differences noted among all three tests. The participants had higher SMM values at retention than at post-measurement. Decreases in the STEPS and VPA were observable during the course of the study, with significant post hoc differences noted between all the measurements.

**Figure 3:**
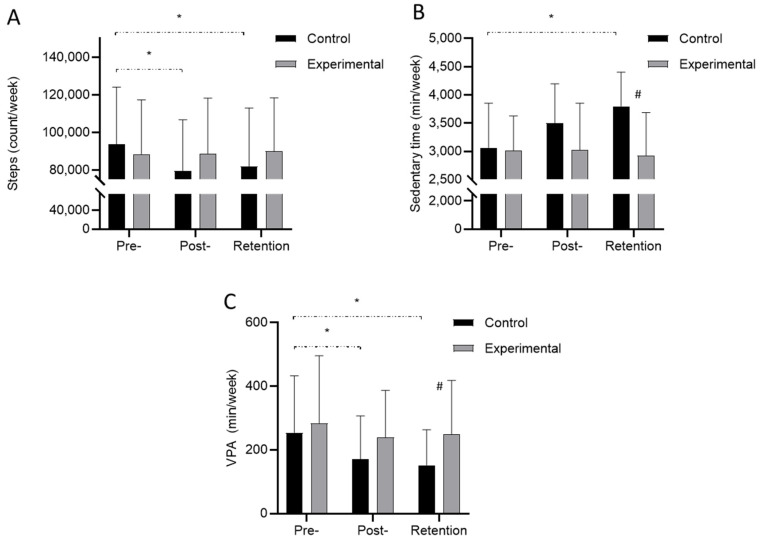
Descriptive statistics (data are presented as means + standard deviations) for study groups ((**A**)—step counts, (**B**)—sedentary time, (**C**)—vigorous physical activity) and significant post hoc differences for variables with significant ANOVA time × group interaction effects (* indicates significant within-group differences at *p* < 0.05, # indicates significant between-group differences for each measurement at *p* < 0.05).

Descriptive statistics and the significance of post hoc differences for the variables showing significant “time × group” interaction effects in the ANOVA are presented in Figure 3. The step count (STEPS) decreased during the course of the study in the C group, with significant post hoc differences noted between the pre- and remaining two measurements (Figure 3A). Furthermore, the ST increased only in the C group (significantly different between pre- and retention), with lower values of ST in the E group than in the C group for retention testing (Figure 3B). Finally, the VPA of the C group decreased during the study course (significant post hoc differences between all measurements), with higher VPA in the E group than in the C group for retention testing (Figure 3C).

## 4. Discussion

With respect to the study aims, several important findings are noted. First, significant positive effects of the PL intervention were found in directly measured indices of PA, specifically, in maintaining the ST, STEPS, and VPA in the E group, with a simultaneous increase in the ST and decreases in the STEP and VPA in the C group. Second, we did not observe significant effects of the PL intervention on the body composition indices of the studied preadolescents. Therefore, our main study hypothesis can be accepted.

### 4.1. Positive Effects on Indices of Physical Activity

Sedentary behavior levels in the E group remained unchanged, whereas those in the C group increased over the course of the study. The increase in sedentarism in the C group is likely associated with school-related and seasonal factors, such as increased homework demands, increased winter months, and reduced daylight hours, all of which contribute to a general increase in sedentary behavior. Indeed, it is well documented that shorter days and colder weather during the winter months limit opportunities for outdoor PA and promote more sedentary time, whereas academic pressures further reduce the time available for movement [55,56,57]. This trend was clearly evident in the C group.

On the other hand, the findings also suggest that well-designed interventions can help mitigate this seasonal decline in PA [56,57]. It seems that this actually occurred in the E group. Most likely, the implemented intervention helped students in the E group develop awareness of the importance of reducing sedentary behavior and provided them with practical strategies to incorporate more PA into their daily lives. As a result, they were able to maintain stable levels of ST despite seasonal and academic factors that typically promote increased sedentariness during the school years. Considering the concept of applied intervention, this outcome highlights the importance of educational approaches that not only increase knowledge but also motivate behavioral change and strengthen children’s ability to manage their own habits, representing the core principles of PL.

The intervention had a significant effect on PA, as measured by the step count. Once again, the results revealed that the step count was maintained over the observed school year in the E group, whereas a decrease was evident in the C group. Most likely, education was effective in encouraging children to engage in more active use of their free time, which plays a key role in preventing sedentary lifestyles and associated health risks. This is consistent with the findings of previous studies in which PL-based interventions were effective in maintaining cardiovascular endurance in adolescents during the COVID-19 pandemic [36]. With respect to this specific variable (STEP), it must be highlighted that throughout the intervention, the children were taught a broader understanding of PA, highlighting the fact that PA includes not only sports but also everyday movements such as walking to school, practicing, or engaging in leisure time. In other words, the intervention aimed not only to increase awareness of the importance of daily movement but also to provide children with practical strategies to integrate PA into daily routines, which together resulted in positive outcomes in the E group.

Consistent with the previous discussions on sedentary behavior and step count, the results indicated that VPA levels in the E group were maintained, whereas a significant decline in VPA was observed in the C group over the course of the study. Although the intervention did not result in an increase in VPA in the E group, considering the previously described (negative) seasonal and school-year effects on PA in children, the observed maintenance of VPA can still be regarded as a positive effect of the intervention [58,59]. The discussed factors that negatively influence PA likely contributed to the decline in VPA in the C group. However, it appears that the PL-based intervention helped maintain levels of VPA over the course of the study in the E group. Therefore, although the intervention did not produce a measurable increase in PA indices, the retention of VPA and step count in the experimental group, compared to the notable declines observed in the control group, represents a meaningful outcome, particularly during a school year period marked by seasonal declines in PA. From an ecological perspective, maintaining baseline levels of VPA and daily steps aligns with public health goals, as even the prevention of decline can support long-term adherence to PA guidelines in children.

One could question the lack of effects in MVPA, but this is probably a result of the classification of accelerometer-measured data. Specifically, the lack of significant effect in MVPA, despite observed differences in VPA and step count, may be partially explained by the way physical activity intensities are classified in accelerometry-based analysis [60]. In brief, MVPA encompasses a broad range of intensities, and it is possible that changes within the upper end (i.e., VPA) were not substantial enough to shift overall MVPA values. Additionally, small fluctuations in light or moderate activity may have offset gains in VPA, resulting in a net neutral effect on the MVPA category.

Collectively, one of the goals of the PL educational content was to highlight that physical activity does not require access to specialized equipment, organized sports, or formal training facilities (please see Materials and Methods for details). As professionals in sport and exercise but also parents themselves, the authors of the educational materials were well aware that the absence of structured physical exercise programs, equipment, and facilities is often overstated as a barrier to PA in all age groups, particularly among children. Indeed, this problem is also highlighted in the literature [61,62]. To address this misconception, the educational video materials and accompanying instructions consistently encouraged children to engage in simple, self-directed forms of movement, such as active play, running, jumping, or stair climbing, activities that can be performed in a variety of environments, including at home or outdoors. Using this approach, the intervention aimed to “demystify” exercise and promote the idea that PA is both accessible and adaptable.

A similar approach was previously found to be effective in adolescents, where the authors confirmed the effects of PL online education on cardiovascular endurance during the COVID-19 pandemic [36]. Furthermore, an 8-week intervention study based on self-determination theory and focused on developing knowledge, motivation, and physical competence (a model closely aligned with our PL-based intervention) resulted in increased VPA levels in children aged 11–13 years [63]. In general, it aligns closely with the foundational concept of PL as defined by Whitehead, who described it as “the motivation, confidence, physical competence, knowledge and understanding to value and take responsibility for engagement in physical activities for life” [64]. Taken together, these findings suggest that PL-based education likely enhanced children’s confidence and perceived autonomy in remaining active, which ultimately led to the results we presented and discussed thus far.

### 4.2. No Significant Effect on Body Composition

As body composition was evaluated as a secondary, exploratory outcome in this study, the lack of significant changes was not unexpected. Several factors may explain this outcome. First, and perhaps most importantly, this result can be attributed to the fact that diet plays a dominant role in regulating body composition in the pediatric population. PA alone, especially when not accompanied by changes in dietary habits, is often insufficient to produce noticeable alterations in body composition [65]. Moreover, it is important to highlight that at this age, children typically do not have autonomy over food choices and intake, relying instead on parental dietary practices, household food availability, and school meal offerings [66,67,68]. Meanwhile, in our study, only two videos addressed nutrition, without direct parental involvement or changes in food environment.

Specifically, a study involving 596 children aged 3–12 years and their parents revealed that parental factors (e.g., parental BMI, dietary styles, and the overall family environment) were significantly associated with children’s BMI and nutritional status [67]. Similarly, a systematic review demonstrated that healthier parenting styles and structured, controlled dietary practices can substantially contribute to improved body composition outcomes in children aged 4–12 years [68]. Similarly, in their systematic review of nutrition and physical activity interventions, Tomayko and colleagues (2021) emphasized that parental involvement is a key factor in improving body weight and preventing obesity in children within this age group [69]. Therefore, although the educational component of our intervention included some nutritional information and evidently positively influenced PA, its impact on body composition was limited simply because of the lack of actual changes in children’s eating behavior.

The second probable reason for the absence of significant effects on children’s body composition may be the insufficient duration of the intervention. For example, a recent systematic review and meta-analysis involving a sample of 20,462 children aged 5–10 years reported that interventions longer than 12 weeks (3 months) generally yield better and more sustainable outcomes in reducing BMI and improving body composition than shorter interventions do [70]. Finally, we should not underestimate the natural variability and dynamic changes in anthropometric measures that occur in children aged 9–11 years. This period is characterized by rapid growth and prepubertal development. During this stage, increases in both fat mass and lean mass are typical and can vary widely among individuals, influenced by genetics, maturation rates, and hormonal changes [71]. As a result, normal growth-related changes may override or mask the effects of short-term interventions. Together, these factors could influence the effects of the PL intervention as well, simply by making it more difficult to detect significant shifts in body composition attributable to the intervention alone.

### 4.3. Limitations and Strengths

This study has several limitations that should be acknowledged. First, although the intervention was standardized across all participants in the E group, it was delivered by different teachers who taught PE for different classes. Next, because of the specifics of the school curriculum, classes and not children were divided into E and C groups. This may have introduced variability in delivery quality and engagement (among PE teachers and children), potentially influencing the study outcomes. However, due to the very limited number of classes (four in total), cluster effects could not be modeled and standard errors may therefore be underestimated. A limitation of the study is the use of BIA equations (Tanita TBF-300), which are not specifically validated for children aged 9–11, and for whom region- and ethnicity-specific standards are still lacking. However, since our focus was on within-subject changes rather than absolute comparisons, the impact on our findings is likely limited. Additionally, the study did not include certain important sociocultural variables (i.e., parental control, neighborhood safety, and the home environment) that could potentially moderate children’s PA behaviors and influence the final results. Also, this study was performed in a specific geographic region with a mild climate, which may limit the generalizability of the findings to areas with unfavorable weather conditions. Additionally, we cannot ignore the fact that teachers were not strictly formally educated and trained on applied interventions, and that limited fidelity monitoring may have introduced variability in intervention delivery across classes. Furthermore, the quasi-experimental group allocation without randomization reduces internal validity and limits the generalizability of the findings. Further studies should simultaneously follow eventual changes in PL facets and their influence on changes in health indices in children. From our perspective, the most significant limitation is the study’s time frame, which covered only one school year rather than a full calendar year. This design excluded important seasonal variations, particularly summer months, which are known to influence PA in children, particularly those living in Mediterranean regions.

Despite these limitations, this study also has several notable strengths. First, this study is among the first to examine a PL-focused educational intervention in early school-aged children via a pre/post/retention design. This allowed us to assess the immediate and sustained effects. Second, PA was measured directly, providing objective and reliable data rather than relying on subjective reports, which are prone to bias. Third, all assessments were conducted by the same group of trained examiners, ensuring consistency in testing procedures and reducing measurement error across time points.

## 5. Conclusions

This study demonstrates that a PL intervention, when embedded within standard PE, can effectively help stabilize sedentary behavior and maintain indices of PA in children during the school year. These findings are particularly relevant given that seasonal and school-related factors often contribute to declining activity levels. It is of particular importance to note that the applied intervention used low-cost, scalable, and curriculum-aligned strategies, making it a potentially practical approach for real-world educational settings.

No specific effects were evidenced in body composition. This may be due to several factors: the absence of a structured nutritional component, the relatively short intervention duration, and the natural variability associated with growth and development in this age group. Additionally, the lack of significant changes may reflect limitations in the precision of body composition measurement tools used in this population.

Importantly, the study’s conclusions are constrained by certain limitations. These include variability in intervention delivery due to differences in teacher training and fidelity, the quasi-experimental design without randomization, and the small number of clusters (classes). These factors could reduce the internal validity and limit the generalizability of the findings to other populations and educational contexts.

Despite these limitations, the study contributes original evidence supporting the behavioral impact of PL-based educational interventions on maintaining children’s PA. Future research should employ randomized controlled designs, include formal fidelity monitoring procedures, and extend intervention duration to account for seasonal variation. Moreover, incorporating broader contextual and sociocultural variables (i.e., parental influence and environmental factors) could provide a more comprehensive understanding of how PL interventions interact with children’s health and development. Also, studies examining the effects of similar interventions on different fitness indicators are warranted.

## Figures and Tables

**Table 1:** Intervention summary.

Component	Description
Duration	3 months (12 weeks)
Frequency	3 PE lessons per week
Total Lessons	36 PE lessons in total
Delivery Format	Short educational videos (3–4 min) integrated into regular PE classes
Number of Videos	12 videos, each repeated 3 times during intervention
Video Themes	2 on PL concepts, 3 on cardiovascular endurance, 5 on motor abilities (strength, power, flexibility, coordination), and 2 on nutrition and healthy habits
Content Focus	Understanding the role of motor abilities in daily life; how to train without structured sport settings
Teaching Tools	Videos, teacher explanations, guided class discussions
Key Practical Strategies	Use of equipment available at home (e.g., ropes, water bottles, carpets); promotion of active transport (e.g., walking, cycling to school)
Control Group Activity	Standard PE curriculum without PL-educational video content

**Table 2:** Main and interaction effects of the repeated-measures factorial ANOVA (F-test) with partial eta squared (η^2^). * denotes statistical significance of *p* < 0.05.

		Intercept	Main Effects			Interaction Effects			
			Group	Gender	Time	Group × Gender	Time × Group	Time × Gender	Time × Group × Gender
Height	F-test	48,688 *	2.11	1.77	740.20 *	7.97 *	1.21	7.97 *	2.11
	η^2^	0.99	0.01	0.01	0.84	0.05	0.01	0.05	0.01
Mass	F-test	1807	3.25	1.36	16.52 *	1.33	0.87	0.77	1.21
	η^2^	0.93	0.02	<0.01	0.11	<0.01	<0.01	<0.01	<0.01
BMI	F-test	3113 *	1.80	0.93	9.89 *	1.31	0.35	1.45	0.05
	η^2^	0.96	0.01	<0.01	0.07	0.01	<0.01	0.01	<0.01
Body fat	F-test	916 *	1.21	1.54	0.19	0.33	0.36	0.39	0.48
	η^2^	0.88	<0.01	0.01	<0.01	<0.01	<0.01	<0.01	<0.01
Skeletal muscle mass	F-test	20,919 *	0.66	6.11 *	12.01 *	2.60	0.79	1.27	1.00
	η^2^	0.99	<0.01	0.05	0.09	0.02	<0.01	0.01	<0.01
STEPS	F-test	1214 *	3.30 *	14.05 *	3.57 *	0.07	4.01 *	0.47	0.99
	η^2^	0.93	0.03	0.15	0.03	<0.01	0.03	<0.01	<0.01
ST	F-test	2700 *	3.11 *	0.52	1.17	0.53	5.34 *	0.46	0.01
	η^2^	0.97	0.03	<0.01	0.02	0.01	0.03	<0.01	<0.01
LPA	F-test	1257 *	0.51	1.17	1.88	0.68	0.59	0.10	0.27
	η^2^	0.93	<0.01	0.01	0.02	<0.01	<0.01	<0.01	<0.01
MVPA	F-test	2194 *	0.01	1.52	1.94	2.44	0.15	0.25	0.61
	η^2^	0.96	<0.01	0.02	0.02	0.03	<0.01	<0.01	<0.01
VPA	F-test	235 *	3.71 *	28.25 *	10.53 *	1.06	4.09 *	1.52	0.35
	η^2^	0.75	0.03	0.25	0.12	0.01	0.04	0.02	<0.01

Legend: STEPS—number of steps per week, ST—sedentary time, LPA—light physical activity, MVPA—moderate-to-vigorous physical activity, VPA—vigorous physical activity.

**Table 3:** Descriptive statistics for the total sample of participants and ANOVA post hoc differences.

	Pre-	Post-	Retention
	Mean ± SD	Mean ± SD	Mean ± SD
Height (cm)	143.43 ± 7.4	144.72 ± 7.26 ^¥^	148.65 ± 7.61 ^¥,£^
Mass (kg)	37.29 ± 10.58	38.04 ± 9.9 ^¥^	39.59 ± 10.2 ^¥,£^
BMI (kg/m^2^)	17.72 ± 3.37	18.02 ± 3.59	17.75 ± 3.39
Body fat (%)	22.98 ± 5.95	23.6 ± 6.49	23.66 ± 7.43
Skeletal muscle mass (%)	43.39 ± 4.07	43.19 ± 3.58	43.95 ± 3.37 ^£^
STEPS (count)	91,605.24 ± 29,755.01	83,511.71 ± 28,483.71 ^¥^	86,681.1 ± 29,937.73 ^¥^
ST (min/week)	3043.47 ± 720.04	3292.58 ± 755.94	3313.48 ± 668.73
LPA (min/week)	2383.58 ± 726.65	2191.31 ± 810.83	2237.23 ± 757.28
MVPA (min/week)	1253.08 ± 336.36	1257.96 ± 350.83	1143.27 ± 329.82
VPA (min/week)	266.41 ± 192.37	194.69 ± 143.28 ^¥^	200.4 ± 168.81 ^¥^

Legend: STEPS—number of steps per week, ST—sedentary time, LPA—light physical activity, MVPA—moderate-to-vigorous physical activity, VPA—vigorous physical activity, ^¥^—significantly different from pre-measurement, ^£^—significantly different from post-measurement.

## Data Availability

The data are available to all interested parties upon reasonable request.

## Supplementary Materials

The video materials of the applied intervention are available here: https://www.youtube.com/playlist?list=PLJl5G3CvzEJ2VHOqFhp2mZreNJrb5XMRv (accessed on 10 January 2026). Supplementary Table S1, including Standardization, Fidelity Monitoring, and Intervention Protocol Details, is available here: https://www.dropbox.com/scl/fi/kongf8klv92g8bul2jmvg/Table-S1-Standardization.docx?rlkey=fl9y9va6r1psnefqz4pcl23ya&dl=0 (accessed on 10 January 2026).

## Abbreviations

The following abbreviations are used in this manuscript:

PA	Physical activity
PL	Physical literacy
PE	Physical education
PF	Physical fitness
LPA	Light physical activity
MVPA	Moderate-to-vigorous physical activity
VPA	Vigorous physical activity
